# Disseminated Bacillus Calmette-Guérin Infection and Immunodeficiency

**DOI:** 10.3201/eid1305.060865

**Published:** 2007-05

**Authors:** Ewa Anna Bernatowska, Beata Wolska-Kusnierz, Malgorzata Pac, Magdalena Kurenko-Deptuch, Zofia Zwolska, Jean-Laurent Casanova, Barbara Piatosa, Jacques van Dongen, Kazimierz Roszkowski, Bozena Mikoluc, Maja Klaudel Dreszler, Anna Liberek

**Affiliations:** * Children's Memorial Health Institute, Warsaw, Poland; † National Institute of Tuberculosis and Lung Diseases, Warsaw, Poland; ‡ University Rene Descartes, Paris, France; § University Medical Center, Utrecht, the Netherlands; ¶ Medical University of Gdańsk, Gdańsk, Poland

**Keywords:** primary immunodeficiencies, disseminated BCG infection, therapeutic guideline, letter

**To the Editor:** Disseminated bacillus Calmette-Guérin (BCG) infection has been noted in patients with primary immunodeficiency. Incidence rates have ranged from 0.06 to 1.56 cases per million vaccinated, and mortality rates have remained at ≈60% (*1*–*7*). Of 946 patients with primary immunodeficiency, including 29 with severe combined immunodeficiencies, diagnosed from 1980 through 2006 at the Children’s Memorial Health Institute in Warsaw, adverse events after BCG vaccination were observed in 16 (*8*,*9*). All 16 were children who had been vaccinated at birth with BCG, Brazilian strain (BioMed, Lublin, Poland).

Four patients with severe combined immunodeficiency showed adverse reactions to BCG. Patient M.K. had mild inflammation at the site of the BCG injection and was successfully treated with rifampin. The patient subsequently received a bone marrow transplant, and 2 months later poor appetite, failure to thrive, and subfebrile condition were noted. Disseminated skin changes (with pus formation in the subcutaneous layer), osteomyelitis, and multiple lesions in the liver were found. A skin biopsy showed tuberculoma formations, which were PCR-positive for *Mycobacterium tuberculosis complex* (Amplified Mycobacterium Tuberculosis Direct [MTD] Test, Gen-Probe, Inc., San Diego, CA, USA) but had negative culture results. Complete recovery, including full immunologic reconstitution, was reached after 12 months of treatment with triple antituberculosis (TB) therapy (rifampin, isoniazid, and ciprofloxacin). Patient M.C., a 6-month-old boy, was admitted to an intensive care unit because of respiratory insufficiency. An unhealed BCG vaccination site was noted. Bronchopulmonary lavage samples were tested for *M. bovis;* positive PCR and culture results led to the diagnosis of disseminated BCG infection. Despite intensive anti-TB therapy, the child died of multiple organ failure. Autopsy showed typical granuloma formations and a hypoplastic thymus, typical for severe combined immunodeficiency. Male patients S.D. and C.G. were admitted to intensive care units at 6 and 8 months of age, respectively, with lymphadenopathy and multiple organ insufficiency. Each boy died of multiple organ failure; postmortem examination found granuloma formation and a hypoplastic thymus in each (*8*).

Eight patients with severe combined immunodeficiency had local adverse events after vaccination with BCG. Inflammation <1 cm in diameter at the vaccination site was observed for all 8. For all except 1, dual anti-TB therapy (rifampin, isoniazid) or monotherapy was successful. For 1 of these patients, anti-TB treatment was stopped 3 months after bone marrow transplant, but increasing inflammation and lymphadenitis appeared 1 month later, with positive PCR and negative culture results for *Mycobacterium* spp. After 12 months of triple anti-TB therapy, this patient fully recovered.

In 2-month-old female patient, W.M., who had interferon-γ–receptor deficiency, axillary lymphadenopathy with normal healing of the vaccination site was noted 7 weeks after BCG vaccination**.** Tuberculous lymphadenitis was diagnosed by histopathologic methods. Despite dual anti-TB therapy and streptomycin administration, the girl died. At autopsy, multiple tuberculous granulomas were found (*5*).

In 4-month-old female patient M.K., who had interleukin-2–receptor deficiency, axillary lymphadenopathy with positive results from *Mycobacterium* typing was noted. Dual anti-TB therapy for 12 months produced good results.

In 7-month-old female patient B.B., axillary lymphadenopathy was noted. Mycobacteria PCR-positive for the *M. tuberculosis* complex were found in the purulent secretion. Despite dual anti-TB therapy, the patient experienced 2 episodes of relapse. After another 2 years of anti-TB therapy, disseminated BCG infection, with pulmonary consequences, developed.

In patient R.C., a 6-month-old boy, osteomyelitis was diagnosed, and delayed healing of the BCG vaccination scar was noted. Investigation of his immunologic status showed no abnormalities. However, because granulomatous inflammation was present in a bone biopsy sample and staining for BCG produced a positive result, triple anti-TB therapy was provided for 12 months, with good results.

The literature describes >200 cases of disseminated BCG infection in patients with primary immunodeficiency (*1*–*7*). The diagnostic difficulties described for 8 of our patients with primary immunodeficiency have been noted by others (*1*–*6*,*8*–*10*). In only 2 cases was the *Mycobacterium* species successfully isolated and identified as *M. tuberculosis* complex, and in 1 case it was the *M. bovis* BCG strain. We propose novel criteria for the diagnosis of disseminated BCG infection in persons with primary immunodeficiency (Table). These criteria have recently been submitted to the European Society for Immunodeficiencies.

**Table:** Suggested diagnostic criteria for disseminated bacillus Calmette-Guérin (BCG) infection in persons with primary immunodeficiency*

Diagnosis	Clinical	Laboratory
Definitive	Systemic symptoms like fever or subfebrile status, weight loss, or stunted growth, and ≥2 areas of involvement beyond the site of BCG vaccination†	Identification of *Mycobacterium bovis* BCG substrain from the patient’s organs by culture and/or standard PCR, as well as typical histopathologic changes with granulomatous inflammation
Probable	Systemic symptoms like fever or subfebrile status, weight loss or stunted growth, and ≥2 areas of involvement beyond the site of BCG vaccination†	Identification of *M. tuberculosis* complex from the organs by PCR, without differentiation of *M. bovis* BCG substrain or other members of the *M. tuberculosis* complex and negative mycobacterial cultures, with the presence of typical histopathologic changes with granulomatous inflammation
Possible	Systemic symptoms like fever or subfebrile condition, weight loss or stunted growth, and ≥2 areas of involvement beyond the site of BCG vaccination†	No identification of mycobacteria by PCR and culture, with presence of typical histopathologic changes with granulomatous inflammation
Exclusion criteria	Any inflammation without typical histopathologic changes, with no isolation of *M. tuberculosis* complex by PCR analysis in patient with primary immunodeficiency	
Differential diagnosis	Severe, long-term inflammation with granuloma formation in patient with primary immunodeficiency	

*Male or female patient with or without genetic confirmation of severe combined immunodeficiency, deficiency of interferon-γ–receptor deficiency, interleukin-12–receptor deficiency, or other primary immunodeficiency with disseminated BCG infection.  †Areas of involvement may include lymph nodes, skin, soft tissues, lungs, spleen, liver, bones.

We believe that patients with severe combined immunodeficiency and any form of mild local changes at the BCG injection site should be given anti-TB therapy, which should be continued until complete immunologic reconstitution occurs after bone marrow transplant. Disseminated BCG infection with regional lymph node involvement needs at least triple anti-TB therapy followed by long-term prophylaxis. Disseminated BCG infection needs anti-TB therapy, including ≥4 anti-TB drugs, until the patient fully recovers.
